# ACKNOWLEDGEMENTS TO REVIEWERS

**DOI:** 10.2174/1570159X2112230822165138

**Published:** 2023-09-25

Bentham Science Publishers would like to thank and appreciate the co-operation from all reviewers for their constructive comments and feedback on the manuscripts submitted to “**Current Neuropharmacology**”. Their efforts have contributed greatly to the high quality and continuous growth of the journal. Given below is the list of reviewers who reviewed articles for the Journal during 2023:

Khaled Abd-Elrahman (Canada)

Miroslav Adzic (Serbia)

Angel Agis-Torres (Spain)

Agorastos Agorastos (Spain)

Umberto Aguglia (Italy)

Gustav Akk (USA)

Marika Alborghetti (Italy)

Angel Alex (India)

Athanasios Alexiou (Austria)

Javed Ali (India)

Davide Amato (Germany)

Antonio F. Ambrosio (Portugal)

George Anderson (Portugal)

Corrado Angelini (Italy)

Nico Antenucci (USA)

Mineyoshi Aoyama (Japan)

Wataru Araki (Japan)

Nadine Attal (France)

Massimo Avoli (Canada)

Ibrahim Badamasi (Nigeria)

Deniz Bagdas (USA)

Gyorgy Bagdy (Hungary)

Giacinto Bagetta (Italy)

Bagshaw Andrew (UK)

Ben A. Bahr (USA)

Malek Bajbouj (Germany)

Rumiana Bakalova (Japan)

Glen Baker (Canada)

Vanessa H. Bal (USA)

Ross J. Baldessarini (USA)

Benedicte Ballanger (France)

Santiago Ballaz (USA)

Cindy Bandala (Mexico)

Matthew Barbaccia (Italy)

Christian Barbato (Italy)

Rita Bardoni (Italy)

Colin Barnstable (USA)

Alasdairm Barr (Canada)

Andrius Baskys (USA)

Agnieszka Basta-Kaim (USA)

Giuseppe Battaglia (Italy)

Michel Baudry (USA)

Luca Becari (USA)

Narasimha Beeraka (Russia)

Tapan Behl (India)

Carmela Belardo (Italy)

David Q. Beversdorf (USA)

Pradeep Bhide (USA)

Ji Bihl (USA)

Pierre Blanchet (Canada)

Arjan Blokland (The Netherlands)

Kenneth Blum (USA)

Istvan Bokkon (USA)

Vadim Bolshakov (USA)

Carla D. Bonan (Brazil)

Luca Bonanni (Italy)

Herlinda Bonilla-Jaime (Mexico)

Ubaldo Bonuccelli (Italy)

Daniel C. Bowers (USA)

Roland Brandt (Germany)

Renan Brito (Brazil)

Natascia Brondino (Italy)

David James Brooks (UK)

Adriaan Bruijnzeel (USA)

Mario Buenrostro-Jauregui (Mexico)

Gary Bukstein Oscar (USA)

Monica Butnariu (Romania)

Jean Lud Cadet (USA)

Zhaolun Cai (China)

Vittorio Calabrese (Italy)

Paolo Calabresi (Italy)

Patrizia Campolongo (Italy)

Francisco Capani (Argentina)

Emily E. Carol (USA)

Simone Carradori (Italy)

Carla Caruso (Argentina)

Patricia Cassina (Uruguay)

Victor Castellanos-Sanchez (Mexico)

Roberta Celli (Italy)

Diego Centonze (Italy)

Sankha Shubhra Chakrabarti (India)

Sumana Chakravarty (India)

Arnob Chakrovorty (India)

Mounia Chami (France)

Indranath Chatterjee (South Korea)

Gang Chen (China)

Yujie Chen (China)

Santina Chiechio (Italy)

Annamaria Cimini (Italy)

Isabel Conceicao (Portugal)

Genaro A. Coria-Avila (Mexico)

Costa Cinzia (Italy)

Luca Cucullo (USA)

Rebecca Cunningham (USA)

Sergio Davinelli (Italy)

Gerry Dawson (UK)

Ladan Dayani (UK)

Domenico De Berardis (Italy)

Augusto de Melo Reis Ricardo (Brazil)

Vito de Novellis (Italy)

Antonio Del Casale (Italy)

Philippe De Deurwaerdere (France)

Diaz-Caneja M. Covadonga (Spain)

Mingxing Ding (China)

Fabio Di Domenico (Italy)

Hailong Dong (China)

Jelena Drulovic (Serbia)

Klaus P. Ebmeier (UK)

Camilla Elefante (Italy)

Guillermo Estivill-Torrús (Spain)

Alex Evers (USA)

Xiaotang Fan (China)

Yong-Gang Fan (China)

Marong Fang (China)

Yuanjian Fang (China)

Liana Fattore (Italy)

Francesco Fazio (Italy)

Jianguo Feng (China)

Jess G. Fiedorowicz (Canada)

Eberval Gadelha Figueiredo (Brazil)

Vittorio Fineschi (Italy)

Patrick Finley (USA)

Marco Fiore (Italy)

Francesco Fornai (Italy)

Francesco Fortunato (Italy)

Franco Javier-Pérez (Mexico)

Valentina Franco (Italy)

Alessandro Frati (Italy)

Marco Aurelio M. Freire (Brazil)

Nicoletta Galeotti (Italy)

Antonio Gambardella (Italy)

Upasana Ganguly (USA)

Fabrizio Gardoni (Italy)

Teresa Gasull (Spain)

Allison Germann (USA)

Goar Gevorkian (Mexico)

Ghezzi Daniele (Italy)

Veronica Ghiglieri (Italy)

Sara Gil-Perotin (Spain)

Sari Goldstein Ferber (Israel)

Benjamin I. Goldstein (Canada)

Jorge Goldstein (Argentina)

Nady Golestaneh (USA)

Ulises Gomez-Pinedo (Spain)

Gong Qiyong (China)

Alejandra Gonzalez-Duarte (Mexico)

Nicholas G. Gottardo (Australia)

Tineke Grent-'t-Jong (UK)

Bruno P. Guiard (France)

Luis Guimaraes (Portugal)

Sinan Guloksuz (The Netherland)

Natalia Gulyaeva (Russia)

Rui-Xian Guo (China)

Xiaoyun Guo (China)

Kathleen M. Gustafson (USA)

Luis Gutierrez-Rojas (Spain)

Kenji Hashimoto (Japan)

Ciria Hernandez (USA)

Ekkehard Hewer (Switzerland)

Raymond Hill (UK)

Hiroaki Hori (Japan)

Bryce Hruska (USA)

Li'An Huang (China)

Peng Huang (China)

Victor Hvingelby (Denmark)

Felice Iasevoli (Italy)

Xiaoyu Ji (China)

Mikhail Kalinichev (Switzerland)

Bozena Kaminska (Poland)

Yuejun Kang (China)

Joshua T. Kantrowitz (USA)

Karatas Hulya (Turkey)

Sukhbir Kaur (USA)

John Kelly (Ireland)

Gopal Khatik (India)

Cecil Kirkland (USA)

Amy Kohtz (USA)

Haruki Koike (Japan)

Peter Kokol (Slovenia)

Dusan Kolar (Canada)

Thomas Kuhn (USA)

Małgorzata Kujawska (Poland)

Robert Kumsta (Denmark)

George Lambrou (Greece)

Doriana Landi (Italy)

Marianna Mazza (Italy)

Peter McCaffery (UK)

Loreta Medina (Spain)

Sandro Da Mesquita (USA)

Vincenzo Micale (Italy)

Euripedes C. Miguel (Brazil)

Mario Miniati (Italy)

Silvia Minozzi (Italy)

Scott J. Moeller (USA)

Sabrina Molinaro (Italy)

Changjong Moon (Republic of Korea)

Michele Morari (Italy)

Thomas Mueller (Germany)

Andrea Negro (Italy)

Kathleen Nicholls (Australia)

Marco Nicola Di (Italy)

Ferdinando Nicoletti (Italy)

Guangjun Nie (China)

Seth D. Norrholm (USA)

Serena Notartomaso (Italy)

Lena Oestreich (Australia)

Kinji Ohno (Japan)

Dominic Oliver (UK)

Ignasi Oliveras (Spain)

Filiz Onat (Turkey)

Elza Othman (Malaysia)

Fernando P. Dominici (Argentina)

Enza Palazzo (Italy)

Konstantinos Palikaras (Greece)

Davide. Papola (Italy)

Raul G. Paredes (Mexico)

Davide Pareyson (Italy)

Lucilla Parnetti (Italy)

Massimo Pasquini (Italy)

Grazia Maria Giovanna Pastorino (Italy)

Francesco Patti (Italy)

Aleksandra M. Pavlovic (Serbia)

Piero Pavone (Italy)

Jaroslav Pejchal (Czech Republic)

Verena Peters (Germany)

Carla Petrella (Italy)

Vittoria Petruzzella (Italy)

Mauro Pettorruso (Italy)

Marco Peviani (Italy)

Simona Pichini (Italy)

Stefano Pieretti (Italy)

Giuseppe Pignataro (Italy)

Pineda-Farias Jorge (Mexico)

Antonio Pisani (Italy)

Emily M. Pitzer (USA)

Cristoforo Pomara (Italy)

Francesco Pontieri (Italy)

Maurizio Popoli (Italy)

Hershel Raff (USA)

Bianca Raffaelli (Germany)

Ricardo Ramírez-Orozco (Mexico)

Luís Rato (Portugal)

Eric A. Reits (The Netherland)

Leandro Ribeiro (Brazil)

Eduardo Rivera-Mancilla (México)

Emmanouil Rizos (Greece)

Laura Rizzi (Italy)

Gianluca Rosso (Italy)

Pamela Rosso (Italy)

Speranza Rubattu (Italy)

Arnold E. Ruoho (USA)

Emilio Russo (Italy)

Roberto Russo (Italy)

Juan C. Saez (Chile)

Marco Salvetti (Italy)

Carmen Sandi (Switzerland)

Marco Scarselli (Italy)

Fabrizio Schifano (UK)

Rudy Schreiber (The Netherland)

Luciana Scotti (Brazil)

Jan Senderek (Germany)

Serafini Gianluca (Italy)

Giulia Serra (USA)

Cinzia Severini (Italy)

Yuan Shi (China)

Paulo R. Shiroma (USA)

Dikoma C. Shungu (USA)

Branca M. Silva (Portugal)

Alessio Simonetti (Italy)

Kirsten Simonsen (Denmark)

Sachin Singh (India)

Varinder Singh (India)

David Smith (USA)

Marta Sochocka (Poland)

Claudia Sommer (Germany)

Fabio Sonvico (Italy)

Edoardo Spina (Italy)

George Stefano (USA)

Natalia Stefanova (Russia)

Robert Steinbach (Germany)

Maksim Storozhuk (Ukarine)

Stephen M. Strakowski (USA)

Teresa Summavielle (Portugal)

Delin Sun (USA)

Xuejun Sun (China)

Takefumi Suzuki (Japan)

Toyofumi Suzuki (Japan)

Mariusz Swiader (Poland)

Rajeev Taliyan (India)

Feng Tao (USA)

Jana D. Tchekalarova (Bulgaria)

Prashant Tiwari (India)

Nicolas Tournier (France)

Werner Treptow (Brazil)

Eugenia Trushina (USA)

Georgios Tsivgoulis (USA)

David E. Vance (USA)

Bhavesh Variya (India)

Rene L. Vidal (Chile)

Dolores Vina (Spain)

XiaoPing Wang (China)

Masahiko Watanabe (Japan)

Cheryl H. Waters (USA)

Aron Weller (USA)

Tadeusz Wieloch (Sweden)

Yohannes W. Woldeamanuel (USA)

Chih-Shung Wong (China)

David C. Wright (Canada)

Mingrui Xia (China)

Jianbo Xiao (China)

Zongyi Xie (China)

Haiwei Xu (China)

Kouichi Yamamoto (Japan)

Feng Yan (China)

Pengfei Yang (China)

Jolanta B. Zawilska (Poland)

Xinwen Zhang (China)

Zhao Ke (China)

Jialin Zheng (China)

Alexander Zholos (Ukraine)

Hao Zhou (China)

